# Impact of Video-Based Error Correction Learning for Cardiopulmonary Resuscitation Training: Quasi-Experimental Study

**DOI:** 10.2196/53577

**Published:** 2024-10-03

**Authors:** Yun Wang, Junzuo Fu, Shaoping Wang, Huijuan Wang, Wei Gao, Lina Huang

**Affiliations:** 1 Department of Anesthesiology, Shanghai General Hospital, Shanghai, China; 2 Department of Gastroenteroscopy, Shanghai General Hospital, Shanghai, China

**Keywords:** video-based error correction, video-prompting, cardiopulmonary resuscitation training, anesthesiology resident, quasi-experimental study, anesthesiology, cardiopulmonary, cardiopulmonary resuscitation, training, video, learning, residents, CPR training, CPR, video prompting

## Abstract

**Background:**

Video-based error correction (VBEC) in medical education could offer immediate feedback, promote enhanced learning retention, and foster reflective practice. However, its application in cardiopulmonary resuscitation (CPR) training has not been investigated.

**Objective:**

The objective of this study is to assess whether the VBEC procedure could improve the training performance of CPR among anesthesiology residents.

**Methods:**

A quasi-experimental study was conducted among anesthesiology residents between December 2022 and April 2023. Primary outcomes included a posttraining knowledge test and practical assessment scores. Secondary outcomes included the number of residents who correctly conducted CPR at each step, the rate of common mistakes during the CPR process, and the self-assessment results. A total of 80 anesthesiology residents were divided into a VBEC group (n=40) and a control group (n=40). The VBEC group underwent a 15-minute VBEC CPR training, whereas the control group underwent a 15-minute video-prompting CPR training.

**Results:**

The posttraining knowledge test score of the VBEC group was significantly higher than that of the control group (73, SD 10.5 vs 65.1, SD 11.4; *P*=.002). The residents in the VBEC group had lower error rates in “failure to anticipate the next move” (n=3, 7.5% vs n=13, 32.5%; *P*=.01) and “failure to debrief or problem solve after the code” (n=2, 5% vs n=11, 27.5%; *P*=.01), as well as better performance in the “secure own safety” step (n=34, 85% vs n=18, 45%; *P*<.001) than those in the control group. The VBEC group showed significantly higher confidence in CPR than the control group (n=?, 62.5% vs n=?, 35%; *P*=.03).

**Conclusions:**

VBEC may be a promising strategy compared to video prompting for CPR training among anesthesiology residents.

## Introduction

### Background

Cardiac arrest remains a significant health care burden despite substantial improvements in survival rates in the past several decades [1]. High-quality cardiopulmonary resuscitation (CPR) is the key link in the chain of survival, and successful CPR may increase the survival rate of patients experiencing cardiac arrest [2,3]. For anesthesiology residents, the likelihood of administering CPR is high, and thus CPR training is of utmost importance [4]. Unfortunately, due to limited training quality and efficiency, the overall quality of CPR remains low, which may further lead to undesired outcomes in clinical practice [5,6]. Thus, CPR instruction modalities are needed to improve the quality of CPR training of anesthesiology residents to maximize their performance.

Video prompting has been shown to be an effective instructional method in the acquisition of a variety of skills, such as vocational. By providing step-by-step guidance and immediate feedback, video prompting can facilitate the development of the necessary skills and confidence in learners required to perform effectively a task [7-9]. Furthermore, emerging evidence suggests that the additional procedure of video-based error correction (VBEC), during which participants who fail to complete a step correctly are interrupted and shown the video prompt again within a short time, can promote skill acquisition more efficiently [10]. VBEC in medical education could offer immediate feedback, promote enhanced learning retention, and foster reflective practice. It allows learners to quickly recognize and correct mistakes, aiding in the memorization of correct procedures through visual demonstration. This method also encourages learners to observe their actions critically, supporting self-improvement and deeper understanding. By providing accessible and consistent instructional content, it ensures equitable learning opportunities. Therefore, introducing the VEBC procedure into CPR training performed with video prompting may enhance the residents’ learning efficiency and performance.

### Objective

In this study, we hypothesized that VBEC would improve the CPR skills of anesthesiology residents.

## Methods

### Study Design and Participants

This quasi-experimental study was conducted in the Simulation Teaching Center of Hospital among anesthesiology residents between December 2022 and April 2023. Participants who met all of the following criteria were included: (1) ≥18 years old; (2) anesthesiology residents; (3) attended CPR training sessions; and (4) primary skill levels for CPR. Residents in elective rotations were excluded.

### Ethical Considerations

Informed consent was obtained from all residents. Ethical approval was received from the Ethics Committee of Shanghai General Hospital (approval #2022KY115). Written informed consent was obtained from all participants.

### Intervention

All residents received a web-based pretest consisting of knowledge and practical assessment based on the Resusci Anne trainer (Laerdal Medical Corporation), with the practical assessment involving the execution of the complete steps of CPR on a manikin. Subsequently, all residents attended a didactic lecture using PowerPoint (Microsoft Corporation) on the American Heart Association (AHA) Guidelines for CPR and Emergency Cardiovascular Care (ECC) [11] and watched a standard CPR video. Then, the residents were divided into VBEC and control groups in the 1:1 ratio.

The training process for the VBEC group was a 15-minute video error correction segment. There were ten CPR video segments with errors in the steps, after which trainees discussed and corrected the errors and practiced the correct steps on a manikin (Multimedia Appendix 1). At the end of each video clip, the trainer announced, “Okay, let’s discuss this step.” The residents were then given 30 seconds to point out the mistake in the video and perform that step on the Resusci Anne trainer in the correct way. This procedure was repeated for all 10 video clips. Further, all residents received 30-minute hands-on standard CPR training, during which they were asked to perform a complete CPR procedure on the Resusci Anne trainer.

The training process for the control group involved a 15-minute video reinforcement segment. There were ten correct CPR step-by-step video segments, after which trainees were asked to practice the correct steps on a manikin following each segment. Then they were asked to perform that step on the Resusci Anne trainer, one at a time. A 30-minute hands-on standard CPR training, the same as that in the VBEC group, was conducted after the video learning in the control group.

At the end of lessons, all residents were asked to independently complete the web-based knowledge test and practical assessment, with the practical test involving performing the entire CPR process on a manikin. The final practical test of each participant was videotaped for evaluation. Finally, all residents completed a web-based questionnaire for self-assessment regarding their performance and satisfaction in this training.

### Outcomes and Measurement

Primary outcomes included the posttraining knowledge test and practical assessment scores. Secondary outcomes were the number of residents who correctly conducted CPR at each step, the rate of common mistakes during the CPR process, and the self-assessment results of the residents after the training.

The practical assessment score was the on-target chest compressions (CCs) indicated by the Resusci Anne trainer, that is, the number of CCs meeting the minimum performance metrics for both CC depth and CC rate. The performance and mistakes of the residents were evaluated by both the Resusci Anne trainer and the human trainers, primarily considering the correct steps of high-quality CPR and the number of common mistakes.

### Statistical Analysis

Data were analyzed using SPSS (version 15; SPSS Inc). Percentages with numbers in parentheses are used to present categorical data. Continuous data are expressed as mean (SD). Continuous variables were compared using the Student 2-tailed *t* test. Categorical variables were compared with the *χ*^2^ test. A 2-sided *P* value <.05 was considered to indicate a statistically significant difference.

## Results

### Baseline Characteristics

**Figure 1.**
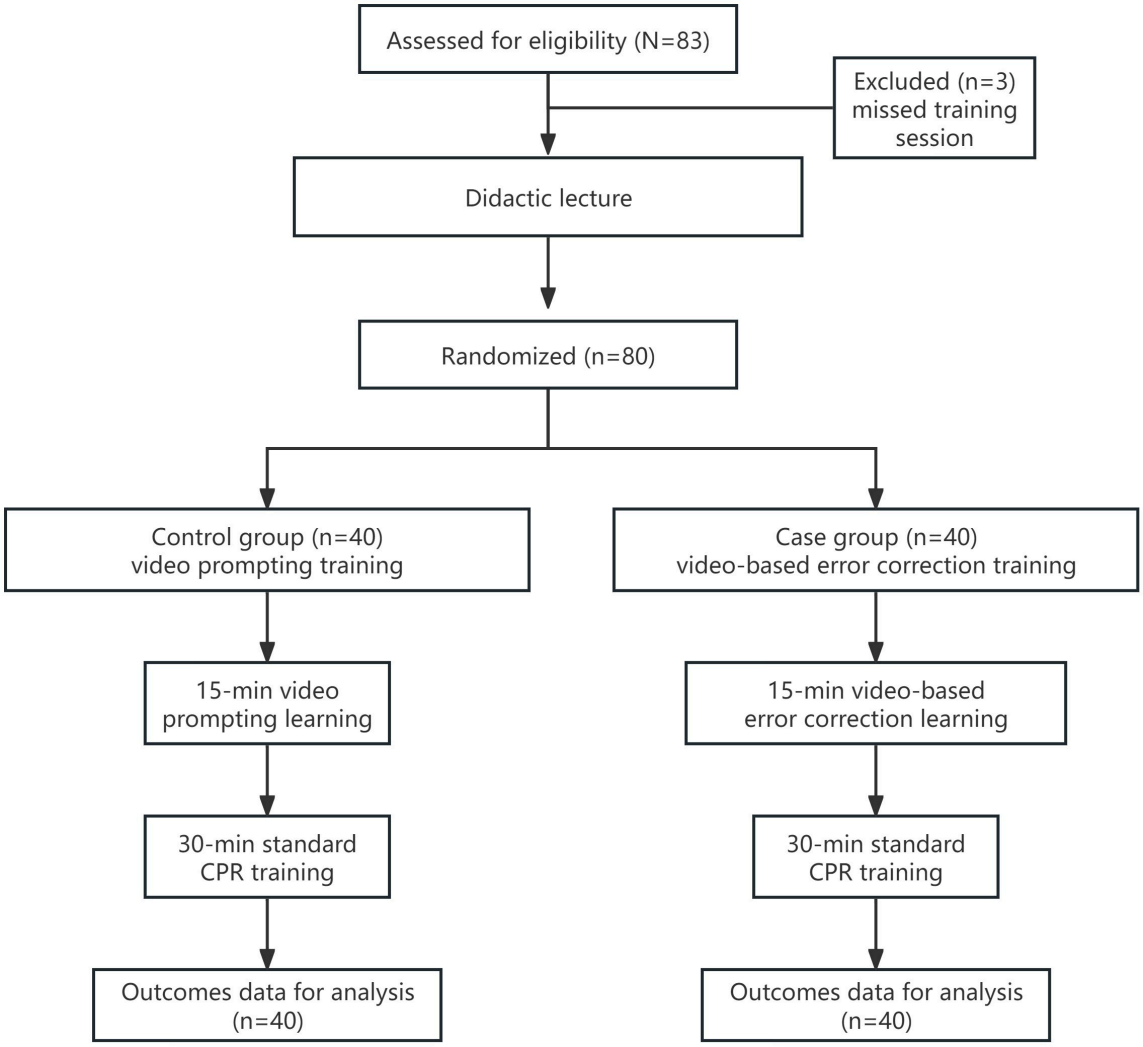
Study flowchart. CPR: cardiopulmonary resuscitation.

Of 83 eligible residents, 80 were subjected to analysis as 3 residents missed part of the training session (Figure 1). The baseline characteristics of the residents are presented in Table 1.

**Table 1.** Baseline characteristics (n=80).

Characteristics	VBEC^a^ (n=40)	Control (n=40)	*P* value
Age (year), mean (SD)	28 (5)	27 (5)	.45
Male, n (%)	20 (50)	22 (55)	.83
Doctors with postgraduate degrees, n (%)	22 (55)	24 (60)	.66

a VBEC: video-based error correction.

### Primary Outcome Results

The pretraining theoretical score (63.9, SD 10.7 vs 63.4, SD 11.6; *P*=.85) and practical scores were comparable between the two groups (81.5 , SD 8.5 vs 82, SD 11.7; *P*=.82). The mean posttraining theoretical score of the VBEC group was significantly higher than that of the control group (73, SD 10.5 vs 65.1, SD 11.4; *P*=.002). No significant difference in the posttraining practical scores was observed between the two groups (86.9, SD 8.8 vs 86.3, SD 9.9; *P*=.77) (Table 2).

**Table 2.** Knowledge test score and practical assessment score before and after the training between the VBEC^a^ and control groups.

		VBEC(n=40)	Control(n=40)	*P* value	*t *(*df*)	95% CI	Effect size
**Knowledge test score, mean (SD)**							
	Before training	63.9 (10.7)	63.4 (11.6)	.85	0.190 (78)	−4.490 to 5.440	.043
	After training	73 (10.5)	65.1 (11.4)	.002	3.228 (78)	3.028 to 12.772	.722
**Practical assessment score, mean (SD)**							
	Before training	81.5 (8.5)	82 (11.7)	.82	−0.229 (78)	−5.082 to 4.032	.051
	After training	86.9 (8.8)	86.3 (9.9)	.77	0.299 (78)	−3.537 to 4.787	.067

a VBEC: video-based error correction

### Secondary Outcome Results

In the stepwise comparison of CPR performance, performance for the “Secure own safety” step was significantly higher in the VBEC group (85% vs 45%; *P*<.001). Among the common mistakes, residents in the VBEC group showed a lower error rate of “failure to anticipate the next move” (7.5% vs 32.5%; *P*=.01) and “failure to debrief or problem solve after the code” (5% vs 27.5%; *P*=.01) than the control group (Table 3).

**Table 3.** Comparison of correct performance and common mistakes of cardiopulmonary resuscitation between the VBEC^a^ and control groups.

	VBEC, n (%)	Control, n (%)	*P* value
Alert emergency services	34 (85)	36 (90)	.74
Recognize illness/injury	40 (100)	40 (100)	>.99
Secure own safety	34 (85)	18 (45)	<.001
Examine patient	34 (85)	30 (75)	.40
Recovery position	36 (90)	38 (95)	.68
Decide if CPR^b^ should be started	38 (95)	32 (80)	.09
Effective chest compressions	38 (95)	35 (87.5)	.43
Open the airway and check breathing	37 (92.5)	36 (90)	>.99
Give rescue breaths	40 (100)	38 (95)	.49
Check rhythms and resume CPR	39 (97.5)	35 (87.5)	.20
Failure to recognize arrest	2 (5)	3 (7.5)	>.99
Failure to act (or act rationally) and failure to call for help appropriately	2 (5)	8 (20)	.09
Failure to provide effective compressions	2 (5)	5 (12.5)	.43
Failure to provide effective ventilations for the patient	0 (0)	1 (2.5)	>.99
Failure to anticipate the next move	3 (7.5)	13 (32.5)	.01
Failure to debrief or problem solve after the code	2 (5)	11 (27.5)	.01

a VBEC: video-based error correction.

b CPR: cardiopulmonary resuscitation.

### Performance After Training

**Figure 2.**
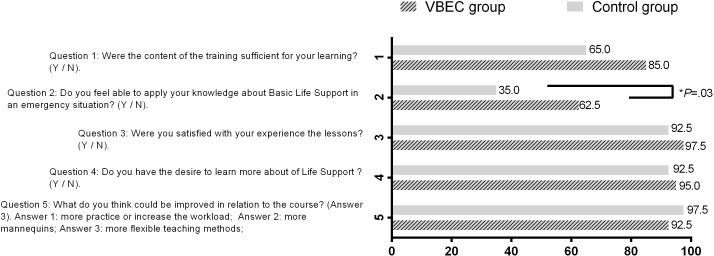
Participants’ positive reactions to activities (%). VBEC: video-based error correction. Asterisk indicates statistical significance.

The residents’ perceptions after the training regarding self-assessment of their performance and related experiences are presented in Figure 2. The residents in the VBEC group had significantly higher confidence in CPR than the control group (n=?, 62.5% vs n=?, 35%; *P*=.03).

## Discussion

### Principal Findings

In this study, it was revealed that VBEC improved the quality of CPR training of anesthesiology residents. Specifically, the VBEC-trained residents had improved posttraining theoretical scores, a lower error rate for common mistakes, and better performance at certain steps of the CPR procedure compared with the control group. The residents who received the VBEC training also showed higher confidence in performing basic CPR than the control group. These findings suggest that VBEC may be applicable in anesthesiology residents’ training.

### Comparison to Prior Work

High-quality CPR can dramatically increase the chances of survival in cardiac arrest. The AHA CPR Guidelines emphasize that to bridge the gap between knowledge and practice, educators should develop a method for improving CPR education [12,13]. Errorless, or near-errorless, learning procedures involve attempting to prevent errors during all teaching sessions [14]. A video prompting technique is to begin teaching with the most assistive prompt to minimize the likelihood of an error [15]. Error correction, on the other hand, involves procedures that are employed following an incorrect response that would increase the probability of a correct response on subsequent trials [16]. Previous research has evidenced the effectiveness of the error correction procedures and has confirmed their benefits [15,17-20]. In this study, we used “error correction learning” as the primary method to improve CPR education and optimize the studying experience. VBEC may provide the learners with the opportunity to think and respond independently to the situation before external instructions are given, which may deepen their understanding of the CPR procedure. This approach may explain the improvement in the posttraining knowledge test among residents in the VBEC group. While some studies argue against the use of error correction since this may cause trainees to pick up incorrect information [21], this study does not support such a conclusion since none of the outcomes of the VBEC group were inferior to those of the control group. On the contrary, the VBEC group had a lower chance of making mistakes that are common in CPR procedures and paid more attention to the rescuer’s safety. Therefore, the VBEC may highlight the error-prone points in standard CPR training. Moreover, Goodson et al demonstrated that VBEC is effective for learners who do not fully benefit from video prompting, as all participants achieved 100% accuracy in task analysis. This outcome was noteworthy, especially since the study included individuals with developmental disabilities [10]. Considering the group-based nature of medical education, this suggests that VBEC could be a valuable tool in the training of medical residents.

### Strengths

No difference was found between the VBEC and the control groups in practical performance after the training. This result may be attributed to the fact that on-target CCs were used to evaluate the practical performance; CC is a muscle memory-based skill that requires more physical practice. However, the 15-minute VBEC or video prompt training plus the 30-minute hands-on training is relatively short for substantially improving the practical performance of the residents. Nevertheless, CC is a central but not the only key part of the whole CPR procedure. Successful CPR in real situations also relies on non-technical skills, such as fluency in the CPR code and physicians’ self-confidence [22,23]. Our results showed that the VBEC group had a lower error rate and higher self-confidence in conducting CPR. More specifically, the fewer “failure to anticipate the next move” in the VBEC group may indicate an enhanced fluency throughout the CPR code. These improvements are essential for the improvement of the overall CPR quality, even with the same CC.

### Limitations

This study is not without limitations. First, the small sample size limits the generalizability of the results, indicating a need for future studies with larger populations to confirm these findings. Second, the brief duration of the testing period suggests the necessity for extended follow-up to fully understand the long-term implications. Third, further investigation is required to explore how VBEC may be applied to improve practical skills such as CC. Moreover, the study’s design constraints, including the absence of power estimation, rigorous randomization, and blinding, hinder our ability to draw definitive causal conclusions, warranting a cautious interpretation of the results.

### Conclusions

In summary, VBEC may be an efficient teaching technique used in CPR training, not only for improving residents’ cognitive performance and self-confidence but also for increasing the rate of completion in providing a fluent CPR sequence with fewer mistakes. However, further research with longer-term follow-up periods is needed to determine whether error correction learning improves long-term outcomes.

## Supplementary material

10.2196/53577 Multimedia Appendix 1 Scenario script of error-correction learning.

## Abbreviations

CC: chest compression
CPR: cardiopulmonary resuscitation
VBEC: video-based error correction
