# Supplementary material for: Impact of Video-Based Error Correction Learning for Cardiopulmonary Resuscitation Training: Quasi-Experimental Study
Source: JMIR Serious Games. 2024 Oct 3;12:e53577. doi: 10.2196/53577 (PMC11466053; doi:10.2196/53577)
Supplement: Multimedia Appendix 1 [file games-v12-e53577-s001.docx]

Scenario script of error-correction learning.

| **Teaching content** | **Error scenario script** |
| --- | --- |
| Recognize illness/injury | CPR is performed on unconscious patients. |
| Pre-CPR judgment and assessment | |
| Secure own safety | CPR is performed on patients at the scene of carbon monoxide poisoning. |
| Examine patient | Call out from a distance of several meters from the patient and use methods such as pinching the nose to determine the patient's response. |
| Call for help | Call "120" for in-hospital patients. |
| Examine patient for respiration and pulse | Wrong body site for palpation of carotid artery; stares at the nose for movement when judging respiration; takes more than 10 seconds to judge respiration and pulse. |
| Recovery position | Compression is applied to a patient lying prone on a loose sofa bed. |
| C-A-B Process | |
| C- Effective chest compressions | Rapid percussion chest compressions. |
| A- Open the airway | Opening the airway by tilting the head and lifting the chin in patients with suspected cervical spine injury. |
| B- Give Rescue Breaths | Artificial breathing without opening the airway; no pinching of the nostrils during artificial breathing. |
| Indications for termination of CPR | |
| Check rhythms and resume CPR | CPR was continued even after the patient developed physical activity. |

CPR: cardio pulmonary resuscitation.
